# 1-(3,4-Dihydroxy­phen­yl)-2-(4-fluoro­phen­yl)ethanone

**DOI:** 10.1107/S1600536808035733

**Published:** 2008-11-08

**Authors:** Xiao-Qing Song

**Affiliations:** a School of Chemical and Material Engineering, Jiangnan University, Lihu Road No. 1800 Wuxi, Wuxi 214122, People’s Republic of China

## Abstract

In the title compound, C_14_H_11_FO_3_, the dihedral angle between the aromatic rings is 69.11 (8)°. An intra­molecular O—H⋯O hydrogen bond is present. Inter­molecular O—H⋯O inter­actions help to establish the packing.

## Related literature

For bond-length data, see: Allen *et al.* (1987 ▶). For background on deoxy­benzoins, see: Li *et al.* (2007 ▶, 2008 ▶).
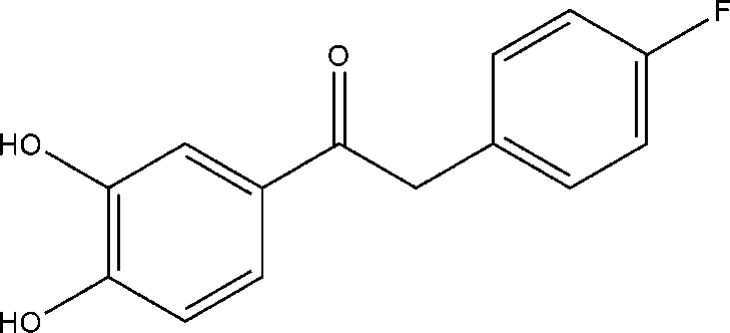


## Experimental

### 

#### Crystal data

C_14_H_11_FO_3_
*M* *_r_* = 246.23
Monoclinic, 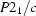

*a* = 8.1640 (16) Å
*b* = 5.9120 (12) Å
*c* = 24.946 (6) Å
β = 105.33 (3)°
*V* = 1161.2 (4) Å^3^
*Z* = 4
Mo *K*α radiation
μ = 0.11 mm^−1^
*T* = 293 (2) K
0.28 × 0.25 × 0.17 mm

#### Data collection

Enraf–Nonius CAD-4 diffractometer
Absorption correction: ψ scan (North *et al.*, 1968 ▶) *T*_min_ = 0.970, *T*_max_ = 0.982
2229 measured reflections
2072 independent reflections
1452 reflections with *I* > 2σ(*I*)
*R*_int_ = 0.030

#### Refinement

*R*[*F* ^2^ > 2σ(*F* ^2^)] = 0.050
*wR*(*F* ^2^) = 0.145
*S* = 1.04
2072 reflections
164 parameters
H-atom parameters constrained
Δρ_max_ = 0.18 e Å^−3^
Δρ_min_ = −0.17 e Å^−3^

### 

Data collection: *CAD-4 Software* (Enraf–Nonius, 1989 ▶); cell refinement: *CAD-4 Software*; data reduction: *XCAD4* (Harms & Wocadlo, 1995 ▶); program(s) used to solve structure: *SHELXS97* (Sheldrick, 2008 ▶); program(s) used to refine structure: *SHELXL97* (Sheldrick, 2008 ▶); molecular graphics: *SHELXTL* (Sheldrick, 2008 ▶); software used to prepare material for publication: *SHELXL97*.

## Supplementary Material

Crystal structure: contains datablocks global, I. DOI: 10.1107/S1600536808035733/hb2831sup1.cif

Structure factors: contains datablocks I. DOI: 10.1107/S1600536808035733/hb2831Isup2.hkl

Additional supplementary materials:  crystallographic information; 3D view; checkCIF report

## Figures and Tables

**Table 1:** Hydrogen-bond geometry (Å, °)

*D*—H⋯*A*	*D*—H	H⋯*A*	*D*⋯*A*	*D*—H⋯*A*
O1—H1*A*⋯O2	0.82	2.28	2.690 (2)	111
O1—H1*A*⋯O2^i^	0.82	2.16	2.876 (3)	146
O2—H2*B*⋯O3^ii^	0.82	1.92	2.744 (2)	178

Symmetry codes: (i) 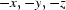; (ii) 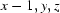.

**Fig. 1.**
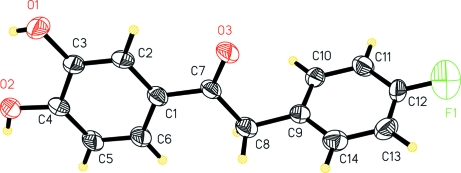
The molecular structure of (I) showing 30% probability displacement ellipsoids for the non-hydrogen atoms.

## supplementary crystallographic information

### Comment

Doxybenzion derivatives play an important role in organic chemistry (Li *et al.*, 2007; Li *et al.*, 2008). In the title compound, (I) (Fig. 1), the bond lengths and angles are within normal ranges (Allen *et al.*, 1987). The dihedral angle between the least-squares planes of the two benzene rings is 69.11 (8) °.   In the crystal, O—H···O hydrogen bonds (Table 1) help to establish the packing.

### Experimental

Pyrocatechol (0.050 mol) and 2-(4-fluorophenyl)acetic acid (0.050 mol) were dissolved into freshly distilled BF_3_Et_2_O under argon. The mixture was stirred at room temperature and then poured in an ice bath. The resulting mixture was extracted with ethyl acetate, and the organic layer was washed with aq. dried (Na_2_S_1_O_4_), and evaporated. The white deposits precipitated were separated from the solvents by filtration. They were washed with aqueous saturated Na_1_H_1_C_1_O_3_ twice. The solid was dissolved in acetone (15 ml) and stirred for about 10 min to give a clear solution. After keeping the solution in air for 10 d, colorless blocks of (I) were formed at the bottom of the vesssl on slow evaporation of the solvent.They were collected, washed three times with acetone and dried in a vacuum desiccator using CaCl_2_. The compound was isolated in 90% yield.

### Refinement

All the H atoms were positioned geometrically (C—H = 0.93–0.96 Å, O—H = 0.82Å) and refined as riding, with *U*_iso_(H) = 1.2*U*_eq_(C) or 1.5U_eq_(O).

### Crystal data

**Table:**

C_14_H_11_FO_3_	*F*_000_ = 512
*M**_r_* = 246.23	*D*_x_ = 1.408 Mg m^−^^3^
Monoclinic, *P*2_1_/*c*	Mo *K*α radiation λ = 0.71073 Å
Hall symbol: -P 2ybc	Cell parameters from 25 reflections
*a* = 8.1640 (16) Å	θ = 9–12º
*b* = 5.9120 (12) Å	µ = 0.11 mm^−^^1^
*c* = 24.946 (6) Å	*T* = 293 (2) K
β = 105.33 (3)º	Block, colorless
*V* = 1161.2 (4) Å^3^	0.28 × 0.25 × 0.17 mm
*Z* = 4	

### Data collection

**Table:**

Enraf–Nonius CAD-4 diffractometer	2072 independent reflections
Radiation source: fine-focus sealed tube	1452 reflections with *I* > 2σ(*I*)
Monochromator: graphite	*R*_int_ = 0.030
*T* = 293(2) K	θ_max_ = 25.2º
ω/2θ scans	θ_min_ = 1.7º
Absorption correction: ψ scan(North *et al.*, 1968)	*h* = −9→0
*T*_min_ = 0.970, *T*_max_ = 0.982	*k* = −7→0
2229 measured reflections	*l* = −28→29

### Refinement

**Table:**

Refinement on *F*^2^	Hydrogen site location: inferred from neighbouring sites
Least-squares matrix: full	H-atom parameters constrained
*R*[*F*^2^ > 2σ(*F*^2^)] = 0.050	*w* = 1/[σ^2^(*F*_o_^2^) + (0.0627*P*)^2^ + 0.5485*P*] where *P* = (*F*_o_^2^ + 2*F*_c_^2^)/3
*wR*(*F*^2^) = 0.145	(Δ/σ)_max_ < 0.001
*S* = 1.04	Δρ_max_ = 0.18 e Å^−^^3^
2072 reflections	Δρ_min_ = −0.17 e Å^−^^3^
164 parameters	Extinction correction: SHELXL97 (Sheldrick, 2008), Fc^*^=kFc[1+0.001xFc^2^λ^3^/sin(2θ)]^-1/4^
Primary atom site location: structure-invariant direct methods	Extinction coefficient: 0.019 (3)
Secondary atom site location: difference Fourier map	

### Special details

**Table:**

Geometry. All e.s.d.'s (except the e.s.d. in the dihedral angle between two l.s. planes) are estimated using the full covariance matrix. The cell e.s.d.'s are taken into account individually in the estimation of e.s.d.'s in distances, angles and torsion angles; correlations between e.s.d.'s in cell parameters are only used when they are defined by crystal symmetry. An approximate (isotropic) treatment of cell e.s.d.'s is used for estimating e.s.d.'s involving l.s. planes.
Refinement. Refinement of *F*^2^ against ALL reflections. The weighted *R*-factor *wR* and goodness of fit *S* are based on *F*^2^, conventional *R*-factors *R* are based on *F*, with *F* set to zero for negative *F*^2^. The threshold expression of *F*^2^ > σ(*F*^2^) is used only for calculating *R*-factors(gt) *etc*. and is not relevant to the choice of reflections for refinement. *R*-factors based on *F*^2^ are statistically about twice as large as those based on *F*, and *R*- factors based on ALL data will be even larger.

### Fractional atomic coordinates and isotropic or equivalent isotropic displacement parameters (Å^2^)

**Table:**

	*x*	*y*	*z*	*U*_iso_*/*U*_eq_	
C1	0.5159 (3)	0.4835 (4)	0.08518 (10)	0.0502 (6)	
C2	0.4714 (3)	0.2980 (4)	0.04986 (10)	0.0536 (7)	
H2A	0.5544	0.2229	0.0374	0.064*	
C3	0.3067 (3)	0.2257 (4)	0.03341 (11)	0.0533 (6)	
C4	0.1810 (3)	0.3396 (4)	0.05140 (10)	0.0496 (6)	
C5	0.2243 (3)	0.5232 (5)	0.08571 (11)	0.0609 (7)	
H5A	0.1406	0.5990	0.0977	0.073*	
C6	0.3901 (3)	0.5969 (5)	0.10269 (12)	0.0622 (8)	
H6A	0.4177	0.7224	0.1258	0.075*	
C7	0.6960 (3)	0.5566 (4)	0.10307 (10)	0.0502 (6)	
C8	0.7428 (3)	0.7480 (5)	0.14453 (12)	0.0636 (8)	
H8A	0.6923	0.8862	0.1266	0.076*	
H8B	0.6923	0.7179	0.1749	0.076*	
C9	0.9297 (3)	0.7876 (4)	0.16843 (10)	0.0496 (6)	
C10	1.0273 (3)	0.6323 (4)	0.20441 (11)	0.0543 (7)	
H10A	0.9768	0.5008	0.2129	0.065*	
C11	1.1974 (3)	0.6678 (5)	0.22796 (12)	0.0614 (7)	
H11A	1.2620	0.5620	0.2522	0.074*	
C12	1.2691 (3)	0.8598 (5)	0.21518 (13)	0.0640 (8)	
C13	1.1793 (4)	1.0184 (5)	0.17977 (13)	0.0693 (8)	
H13A	1.2317	1.1486	0.1715	0.083*	
C14	1.0083 (4)	0.9806 (5)	0.15645 (12)	0.0624 (7)	
H14A	0.9450	1.0872	0.1322	0.075*	
F1	1.4375 (2)	0.8958 (4)	0.23816 (10)	0.1039 (7)	
O1	0.2648 (2)	0.0477 (4)	−0.00250 (10)	0.0813 (7)	
H1A	0.1726	−0.0035	−0.0012	0.122*	
O2	0.0199 (2)	0.2558 (3)	0.03311 (8)	0.0615 (5)	
H2B	−0.0421	0.3199	0.0493	0.092*	
O3	0.8048 (2)	0.4626 (3)	0.08579 (8)	0.0650 (6)	

### Atomic displacement parameters (Å^2^)

**Table:**

	*U*^11^	*U*^22^	*U*^33^	*U*^12^	*U*^13^	*U*^23^
C1	0.0430 (13)	0.0574 (15)	0.0503 (14)	0.0101 (11)	0.0126 (10)	−0.0043 (12)
C2	0.0470 (14)	0.0553 (15)	0.0637 (16)	0.0095 (12)	0.0240 (12)	−0.0087 (13)
C3	0.0513 (14)	0.0511 (15)	0.0610 (15)	0.0044 (12)	0.0208 (12)	−0.0110 (13)
C4	0.0426 (13)	0.0552 (15)	0.0528 (14)	0.0084 (11)	0.0157 (11)	0.0012 (12)
C5	0.0429 (14)	0.0747 (19)	0.0676 (17)	0.0135 (13)	0.0190 (12)	−0.0202 (15)
C6	0.0477 (14)	0.0707 (18)	0.0689 (17)	0.0087 (13)	0.0169 (12)	−0.0199 (15)
C7	0.0424 (12)	0.0580 (16)	0.0503 (14)	0.0099 (12)	0.0127 (11)	−0.0033 (12)
C8	0.0495 (14)	0.0680 (18)	0.0720 (18)	0.0114 (13)	0.0139 (13)	−0.0164 (15)
C9	0.0475 (13)	0.0477 (14)	0.0550 (15)	0.0049 (11)	0.0163 (11)	−0.0086 (12)
C10	0.0524 (14)	0.0447 (14)	0.0690 (17)	−0.0029 (12)	0.0216 (12)	0.0032 (13)
C11	0.0513 (15)	0.0593 (17)	0.0721 (18)	0.0063 (13)	0.0137 (13)	0.0068 (14)
C12	0.0458 (14)	0.0609 (17)	0.087 (2)	−0.0100 (13)	0.0207 (14)	−0.0153 (16)
C13	0.075 (2)	0.0485 (16)	0.093 (2)	−0.0114 (15)	0.0380 (17)	−0.0034 (16)
C14	0.0721 (18)	0.0514 (16)	0.0657 (18)	0.0113 (14)	0.0216 (14)	0.0065 (14)
F1	0.0526 (10)	0.0961 (14)	0.157 (2)	−0.0212 (10)	0.0171 (11)	−0.0227 (14)
O1	0.0564 (11)	0.0761 (14)	0.1190 (18)	−0.0081 (10)	0.0364 (11)	−0.0460 (13)
O2	0.0454 (9)	0.0655 (12)	0.0780 (12)	0.0031 (9)	0.0241 (8)	−0.0133 (10)
O3	0.0458 (10)	0.0736 (13)	0.0791 (13)	0.0107 (9)	0.0226 (9)	−0.0201 (10)

### Geometric parameters (Å, °)

**Table:**

C1—C6	1.391 (3)	C8—H8A	0.9700
C1—C2	1.393 (3)	C8—H8B	0.9700
C1—C7	1.483 (3)	C9—C14	1.380 (4)
C2—C3	1.366 (3)	C9—C10	1.380 (3)
C2—H2A	0.9300	C10—C11	1.374 (3)
C3—O1	1.365 (3)	C10—H10A	0.9300
C3—C4	1.397 (3)	C11—C12	1.354 (4)
C4—O2	1.366 (3)	C11—H11A	0.9300
C4—C5	1.369 (4)	C12—F1	1.359 (3)
C5—C6	1.378 (3)	C12—C13	1.362 (4)
C5—H5A	0.9300	C13—C14	1.381 (4)
C6—H6A	0.9300	C13—H13A	0.9300
C7—O3	1.219 (3)	C14—H14A	0.9300
C7—C8	1.512 (4)	O1—H1A	0.8200
C8—C9	1.503 (3)	O2—H2B	0.8200
			
C6—C1—C2	119.2 (2)	C9—C8—H8B	108.3
C6—C1—C7	121.4 (2)	C7—C8—H8B	108.3
C2—C1—C7	119.4 (2)	H8A—C8—H8B	107.4
C3—C2—C1	120.5 (2)	C14—C9—C10	118.0 (2)
C3—C2—H2A	119.8	C14—C9—C8	121.6 (2)
C1—C2—H2A	119.8	C10—C9—C8	120.3 (2)
O1—C3—C2	119.6 (2)	C11—C10—C9	121.4 (2)
O1—C3—C4	120.3 (2)	C11—C10—H10A	119.3
C2—C3—C4	120.1 (2)	C9—C10—H10A	119.3
O2—C4—C5	124.3 (2)	C12—C11—C10	118.6 (3)
O2—C4—C3	116.2 (2)	C12—C11—H11A	120.7
C5—C4—C3	119.5 (2)	C10—C11—H11A	120.7
C4—C5—C6	120.9 (2)	C11—C12—F1	119.0 (3)
C4—C5—H5A	119.6	C11—C12—C13	122.6 (3)
C6—C5—H5A	119.6	F1—C12—C13	118.5 (3)
C5—C6—C1	119.9 (3)	C12—C13—C14	118.2 (3)
C5—C6—H6A	120.1	C12—C13—H13A	120.9
C1—C6—H6A	120.1	C14—C13—H13A	120.9
O3—C7—C1	121.2 (2)	C9—C14—C13	121.2 (3)
O3—C7—C8	120.5 (2)	C9—C14—H14A	119.4
C1—C7—C8	118.3 (2)	C13—C14—H14A	119.4
C9—C8—C7	115.7 (2)	C3—O1—H1A	109.5
C9—C8—H8A	108.3	C4—O2—H2B	109.5
C7—C8—H8A	108.3		
			
C6—C1—C2—C3	−1.2 (4)	C2—C1—C7—C8	−175.8 (2)
C7—C1—C2—C3	179.1 (2)	O3—C7—C8—C9	−8.8 (4)
C1—C2—C3—O1	178.1 (2)	C1—C7—C8—C9	169.9 (2)
C1—C2—C3—C4	0.9 (4)	C7—C8—C9—C14	112.3 (3)
O1—C3—C4—O2	2.9 (4)	C7—C8—C9—C10	−69.3 (3)
C2—C3—C4—O2	−179.9 (2)	C14—C9—C10—C11	0.3 (4)
O1—C3—C4—C5	−177.5 (3)	C8—C9—C10—C11	−178.2 (2)
C2—C3—C4—C5	−0.3 (4)	C9—C10—C11—C12	−0.1 (4)
O2—C4—C5—C6	179.6 (3)	C10—C11—C12—F1	−179.8 (3)
C3—C4—C5—C6	0.0 (4)	C10—C11—C12—C13	−0.3 (4)
C4—C5—C6—C1	−0.4 (4)	C11—C12—C13—C14	0.3 (5)
C2—C1—C6—C5	1.0 (4)	F1—C12—C13—C14	179.9 (3)
C7—C1—C6—C5	−179.4 (3)	C10—C9—C14—C13	−0.2 (4)
C6—C1—C7—O3	−176.9 (3)	C8—C9—C14—C13	178.3 (2)
C2—C1—C7—O3	2.8 (4)	C12—C13—C14—C9	−0.1 (4)
C6—C1—C7—C8	4.5 (4)		

### Hydrogen-bond geometry (Å, °)

**Table:**

*D*—H···*A*	*D*—H	H···*A*	*D*···*A*	*D*—H···*A*
O1—H1A···O2	0.82	2.28	2.690 (2)	111
O1—H1A···O2^i^	0.82	2.16	2.876 (3)	146
O2—H2B···O3^ii^	0.82	1.92	2.744 (2)	178

Symmetry codes: (i) −*x*, −*y*, −*z*; (ii) *x*−1, *y*, *z*.

## References

[bb1] Allen, F. H., Kennard, O., Watson, D. G., Brammer, L., Orpen, A. G. & Taylor, R. (1987). *J. Chem. Soc. Perkin Trans. 2*, pp. S1–19.

[bb2] Enraf–Nonius (1989). *CAD-4 Software* Enraf–Nonius, Delft, The Netherlands.

[bb3] Harms, K. & Wocadlo, S. (1995). *XCAD4* University of Marburg, Germany.

[bb4] Li, H.-Q., Xu, C., Li, H.-S., Xiao, Z.-P., Shi, L. & Zhu, H.-L. (2007). *ChemMedChem*, **2**, 1361–1369. 10.1002/cmdc.200700097 17628869

[bb5] Li, H.-Q., Xue, J.-Y., Shi, L., Gui, S.-Y. & Zhu, H.-L. (2008). *Eur. J. Med. Chem.* **43**, 662–667. 10.1016/j.ejmech.2007.05.013 17624635

[bb6] North, A. C. T., Phillips, D. C. & Mathews, F. S. (1968). *Acta Cryst.* A**24**, 351–359.

[bb7] Sheldrick, G. M. (2008). *Acta Cryst.* A**64**, 112–122. 10.1107/S0108767307043930 18156677

